# Evaluation of a new fluorescence quantitative PCR test for diagnosing *Helicobacter pylori* infection in children

**DOI:** 10.1186/1471-230X-13-7

**Published:** 2013-01-14

**Authors:** Zhiying Ou, Liya Xiong, Ding-You Li, Lanlan Geng, Lixia Li, Peiyu Chen, Min Yang, Yongmei Zeng, Zhenwen Zhou, Huimin Xia, Sitang Gong

**Affiliations:** 1 Department of Gastroenterology, Guangzhou Women and Children’s Medical Center, Guangzhou Medical College, 9 Jinsui Road, Guangzhou, 510623, China; 2 Department of Pediatrics, University of Missouri Kansas City School of Medicine, Division of Gastroenterology, Children’s Mercy Hospital, 2401 Gillham Road, Kansas City, MO, 64108, USA

**Keywords:** Fluorescence quantitative PCR, *Helicobacter pylori*, Gastric mucosa, Gastric fluids, Dental plaques, Children

## Abstract

**Background:**

Numerous diagnostic tests are available to detect *Helicobactor pylori* (H. pylori). There has been no single test available to detect H. pylori infection reliably. We evaluated the accuracy of a new fluorescence quantitative PCR (fqPCR) for H. pylori detection in children.

**Methods:**

Gastric biopsy specimens from 138 children with gastritis were sent for routine histology exam, rapid urease test (RUT) and fqPCR. ^13^C-urea breath test (^13^C-UBT) was carried out prior to endoscopic procedure. Gastric fluids and dental plaques were also collected for fqPCR analysis.

**Results:**

38 children (27.5%) were considered positive for H. pylori infection by gold standard (concordant positive results on 2 or more tests). The remaining 100 children (72.5%) were considered negative for H. pylori. Gastric mucosa fqPCR not only detected all 38 H. pylori positive patients but also detected 8 (8%) of the 100 gold standard-negative children or 11 (10.7%) of the 103 routine histology-negative samples. Therefore, gastric mucosa fqPCR identified 46 children (33.3%) with H. pylori infection, significantly higher than gold standard or routine histology (P<0.01). Both gastric fluid and dental plaque fqPCR only detected 32 (23.2%) and 30 (21.7%) children with H. pylori infection respectively and was significantly less sensitive than mucosa fqPCR (P<0.05) but was as sensitive as non-invasive UBT.

**Conclusions:**

Gastric mucosa fqPCR was more sensitive than routine histology, RUT, ^13^C-UBT alone or in combination to detect H. pylori infection in children with chronic gastritis. Either gastric fluid or dental plaque PCR is as reliable as ^13^C-UBT for H. pylori detection.

## Background

*Helicobacter pylori* (H. pylori) is one of the most common chronic bacterial infections in developing countries. The overall prevalence of H. pylori infection in southern China is 44.2% in children and 61.6% in symptomatic patients [1,2]. It is well known that a successful eradication of H. pylori dramatically reduces the rate of recurrence of gastric and duodenal ulcers in affected children. Therefore, an accurate H. pylori test is crucial for initiation of appropriate treatment.

Numerous diagnostic tests are available to detect H. pylori infection and are divided into either invasive (histology, rapid urease test (RUT) and bacterial culture) or non-invasive tests (serology, ^13^C-urea breath test (^13^C-UBT), and stool antigen test) [3]. However, there has been no single test available that can be used as a gold standard to detect H. pylori infection reliably and accurately [4,5]. Bacterial culture of gastric tissues has 100% specificity but sensitivity is low. Histological exam and RUT provided excellent diagnostic sensitivity and specificity of approximately 90–95% but the detection rate for H. pylori decreased in the presence of bleeding peptic ulcers or gastric atrophy [6,7]. The ^13^C-UBT is a safe, noninvasive and reliable method for diagnosing H. pylori infection in adults but is less accurate for the diagnosis of H. pylori infection in infants and young children [8]. The accuracy of non-invasive stool antigen test is excellent and comparable to that of other methods [9]. However, a wide range for sensitivity and specificity for stool antigen test was reported depending upon types of commercial test being used, cut-off value, treatment status and interpretation of weakly-positive results [5,9]. Therefore, it is recommended that concordant results of at least two tests are needed to define the H. pylori infection status in children [5].

PCR-based methods have been shown to be the most reliable method for H. pylori diagnosis [10-13]. We recently developed and validated a new fluorescent quantitative PCR (fqPCR) to detect H. pylori infection. The aim of this prospective study was to evaluate the accuracy of this new fqPCR test for detection of H. pylori infection in children, in comparison to commonly used tests: routine histology, RUT and ^13^C-UBT.

## Methods

### Patients

From July to December 2011, 138 consecutive eligible patients undergoing routinely scheduled endoscopy at Guangzhou Women and Children’s Medical Center (Guangzhou, China) were selected to participate in this study after informed consent was obtained from parents. The institutional Review Board of Guangzhou Women and Children’s Medical Center approved the study protocol. We included any children who had gastrointestinal symptoms and chronic gastritis determined by histology. We excluded children who had taken antibiotics, proton pump inhibitors, H2-antogonists or gastric motility drugs within 4 weeks prior to endoscopy. We also excluded patients who had acute gastrointestinal bleeding within 1 week of endoscopy.

### Endoscopic procedure

Endoscopy was performed by a trained pediatric gastroenterologist. Multiple biopsy specimens were obtained from the antrum of the stomach. Two biopsies were used for RUT, 2 for histology and 2 for fqPCR assay. During endoscopy, 1 ml of gastric fluid was collected for fqPCR. In addition, dental plaque was collected from each patient for DNA extraction and fqPCR prior to endoscopic procedure.

### Routine histologic evaluation

According to standard protocol, antral specimens were fixed in a 10% buffered formalin solution, embedded in paraffin and stained with hematoxylin and eosin and Giemsa for histology and the presence of H. pylori bacteria was determined by two experienced pathologists, who were blinded to all other test results.

### ^13^C-UBT

^13^C-UBT was performed according to a standard protocol included in the diagnosis kit (YouErTe Inc, Beijing, China). After fasting for at least 4 hours, a baseline breath sample was collected. Another breath sample was collected 30 min after oral administration of 75 mg of ^13^C-urea dissolved in 50 ml of water. All the breath samples were analyzed using a single gas isotope ratio mass spectrometer (Yanghe Meidcal Inc., Beijing, China). According to the manufacture’s recommendation, the test was considered positive if the 30 min delta value over baseline value (DOB) exceeded 4.0, which is consistent with the cut-off value used by others [14,15].

### RUT

H. pylori chemical reaction test paper (Kedi Science Developmental Inc., Zhuhai, China) has been used routinely in our hospital with comparable sensitivity and specificity to conventional RUT (CLO-test). Antral biopsy specimens were put in the middle of the test paper at room temperature. According to the manufacture’s recommendation, a color change to red within three minutes was considered positive.

### fqPCR

Bacterial genomic DNA was extracted by using boiling lysis. The dental plaque sample was rinsed in 500 μl of 0.9% NaCl and centrifuged (Eppendorf 5148) at 10,000 x g for 5 min. 50 μl DNA extraction buffer was added into the precipitate. Gastric fluid was centrifuged at 10,000 x g for 5 min, and 50 μl DNA extraction buffer was added into the precipitate. One gastric biopsy specimen was homogenized after adding 50 μl DNA extraction buffer.

Each sample was incubated at 100°C for 10 min in 50 μl DNA extraction buffer containing 50 mM NaOH, 10 mM Tris–HCl (pH 8.0), 1% Triton X-100, 1% NP-40, and 0.5 mM EDTA (pH 8.0). After centrifuged at 15,000 x g for 5 min, 5 microliters of the supernatant was amplified by PCR in 50 μl of 1×PCR buffer containing 200 μM of each deoxynucleoside triphosphate (dNTP), 2 U of *Taq* DNA polymerase, 0.12 μM of TaqMan probe and 0.2 μM of each primer. In order to prevent contamination, we replaced dTTP with dUTP and added 0.5 U of uracil-DNA glycosylase (UDG) to the PCR system. The amplification was performed by using LightCycler 480II (Roche) under the following conditions: incubation at 50°C for 3 min, followed by denaturation at 94°C for 3 min, and 40 cycles of 94°C for 10 s, 55°C for 45 s, and fluorescence collection at 55°C. A final extension was performed at 40°C for 1 min. PCR primers and probe for the target gene UreA were designed by Primer Express 2.0. Forward primer sequence is 5'-GGC TGA ATT GAT GCA AGA AG-3; reverse primers sequence is 5'-GGT ATG CAC GGT TAC GAG TT-3' and TaqMan probe sequence is FAM-5'-TCCC ATC AGG AAA CAT CGC T-3'-TAMRA. Basic Local Alignment Search Tool (BLAST) database searches were performed for primers and probes to find any sequence similarities. No matches with any human or bacterial DNA targets were found for any of the selected primers and probes.

### Gold standard for H. pylori infection status

We defined H. pylori infection status as positive if at least two different tests among histology, RUT and ^13^C-UBT showed concordant positive results [5]. H. pylori-negative status is defined as all negative or just one positive result among all three tests, histology, RUT and ^13^C-UBT.

### Statistical analysis

Statistical analysis was carried out by using SPSS software version 16.0 (SPSS, Inc., Chicago, III). Chi-square and Fisher's exact tests were applied to our analysis. *P* values <0.05 was considered statistically significant.

## Results

### Patient characteristics

A total of 138 children (male 94, mean age of 8.3 years, range 2–14 years) were enrolled in this study. The indications for endoscopy included abdominal pain, nausea, vomiting, anorexia, iron-deficiency anemia, heartburn, hiccups, fullness, anorexia, and acid reflux. All participants had been diagnosed with chronic gastritis confirmed by histology.

### H. pylori infection status according to reference standard and each individual test

As shown in Table 1, 38 children (27.5%) were positive for H. pylori infection according to the gold standard. The remaining 100 children (72.5%) were considered negative for H. pylori. Note that among those, 87 patients were negative in all three tests applied, and 13 patients were positive for just one test (histology: n=0; RUT: n=10; UBT: n=3).

**Table 1:** **Results of various methods and gold standard for detecting*Helicobacter pylori*infection in children with gastritis**

**H. pylori status**	**Gold standard**	**Routine Histology**	**RUT**	^**13**^**C-UBT**	**Gastric mucosal fqPCR**	**Gastric fluid fqPCR**	**Dental plaque fqPCR**
**Positive n (%)**	38 (27.5%)	35 (25.4%)	45 (32.6%)	31 (22.5%)	46 (33.3%)	32 (23.2%)	30 (21.7%)
**Negative n (%)**	100 (72.5%)	103 (74.6%)	93 (77.4%)	107 (77.5%)	92 (66.7%)	106 (76.8%)	108 (78.3%)

RUT: rapid urease test; ^13^C-UBT, ^13^C-urea breath test; fqPCR, florescent quantitative PCR.

Routine histology, RUT and ^13^C-UBT were positive for H. pylori in 25.4%, 32.6% and 22.5% of 138 children, respectively. H. pylori positivity rate was significantly higher with RUT than with histology or ^13^C-UBT (p < 0.01).

Gastric biopsy specimen fqPCR was positive for H. pylori in 46 (33.3%) children, significantly higher than gold standard (p < 0.01). However, both gastric fluid and dental plaque fqPCR only detected 32 (23.2%) and 30 (21.7%) children with H. pylori infection respectively and was significantly less sensitive than gold standard (P<0.05) but as sensitive as ^13^C-UBT.

### Comparison of different assays for H. pylori infection detection using gold standard or mucosa fqPCR as the reference

We conducted validation studies for fqPCR for H. pylori infection. We enrolled 10 H. pylori-negative and 10 H. pylori-positive control patients. Negative controls were those with normal routine histology and negative results on RUT, ^13^C-UBT and stool H. pylori antigen test. Positive controls were tested positive not only on routine histology but also on RUT, ^13^C-UBT and stool H. pylori antigen test. All the H. pylori-positive control samples were positive by fqPCR and all the H. pylori-negative control samples were negative for fqPCR, with 100% sensitivity and 100% specificity (data not shown).

As shown in Table 1, gastric mucosa fqPCR detected 46 children with H. pylori infection. All 38 gold standard positive children were positive for gastric mucosa fqPCR. In addition, 8 (8%) of the 100 gold standard negative children or 11 (10.7%) of the 103 routine histology-negative samples were also positive for gastric mucosa fqPCR. 87 patients with negative results in all three tests were negative for gastric mucosa fqPCR, whereas 8 of the 13 patients with just one positive test were positive for gastric mucosa fqPCR.

Based on the gold standard, the sensitivities of routine histology, RUT, ^13^C-UBT, gastric fluid fqPCR and dental plaque fqPCR were 92.1%, 92.1%, 81.6%, 84.2% and 78.9%, respectively (Table 2). In comparison, the sensitivities of routine histology, RUT, ^13^C-UBT, gastric fluid fqPCR and dental plaque fqPCR were significantly lower at 76.1%, 76.1%, 60.9%, 69.6% and 65.2% respectively if gastric mucosa fqPCR was used as a reference standard. Specificities were between 92% and 100% with gold standard or 89.1% and 100% with mucosa fqPCR. Routine histology and all the fqPCR assays had 100% specificity and 100% positive predictive value (PPV) with either gold standard or mucosa fqPCR. Negative predictive values (NPV) of routine histology, RUT, ^13^C-UBT, gastric fluid fqPCR and dental plaque fqPCR were at 85.2-97.1% with gold standard or 83.2 to 89.2% with mucosa fqPCR.

**Table 2:** Comparison of various methods for diagnosing H. pylori infection in children with gastritis, using gold standard (GS) or mucosal florescent quantitative PCR (mfqPCR) as the reference

	**Routine histology**		**RUT**		^**13**^**C-UBT**		**Gastric fluid PCR**		**Dental plaque PCR**	
Standard	GS	mfqPCR	GS	mfqPCR	GS	mfqPCR	GS	mfqPCR	GS	mfqPCR
Sensitivity %	92.1	76.1	92.1	76.1	81.6	60.9	84.2	69.6	78.9	65.2
Specificity %	100.0	100.0	90.0	89.1	97.0	96.7	100.0	100.0	100.0	100.0
PPV %	100.0	100.0	77.8	77. 8	90.3	90.3	100.0	100.0	100.0	100.0
NPV %	97.1	89.3	96.8	88.2	90.1	83.2	94.3	86.8	92.6	85.2

PPV: positive predictive value; NPV: negative predictive value; RUT: rapid urease test; ^13^C-UBT, urea breath test.

With mucosa fqPCR as reference, the sensitivity and specificity of gastric fluid fqPCR or dental plaque fqPCR are similar to that of ^13^C-UBT (p > 0.05).

### Age distribution of H. pylori in children with gastritis

**Figure 1:**
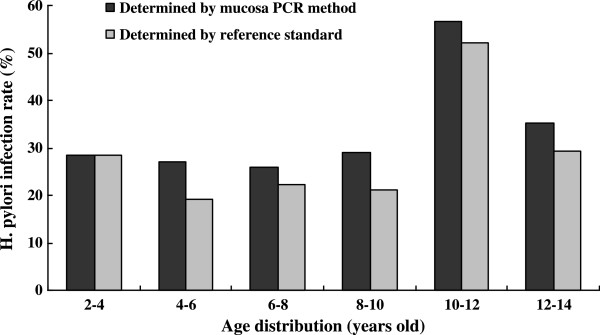
Age distribution of H. pylori infection rates determined by mucosal PCR method and reference standard (positive results concordant on 2 or more tests) in 138 children with gastritis.

As shown in Figure 1, H. pylori infection rate determined by gold standard varied from 16.7% to 50% among different age groups in 138 children with gastritis. In contrast, H. pylori infection rate determined by mucosal fqPCR method is higher and varied from 25.9% to 56.5% among different age groups. H. pylori infection rate is the highest at 10–12 year-old children with gastritis.

## Discussion

We recently developed a new fqPCR for H. pylori detection, using uracil-DNA glycosylase (UDG) [16] in the reaction system to prevent PCR product contamination and effectively eliminate the false positive results. In addition, single closed-tube assay for each sample reduces aerosol contamination. Our new PCR assay is fast and takes less than 3 hours from sample processing to final result.

In this study, we found that gastric mucosa fqPCR is more sensitive than routine histology, RUT, ^13^C-UBT or the commonly used gold standard for detecting H. pylori infection in children with gastritis. Not only did fqPCR detect H. pylori in all gold standard or routine histology positive samples, it also detected H. pylori infection in 11 of the 103 routine histology-negative patients or in 8 of the 100 gold standard-negative patients. To our best knowledge, this is the first report of a novel fqPCR-based assay to establish H. pylori infection in children.

PCR-based techniques have been shown to be more sensitive than conventional methods for H. pylori detection. Lage et al. [17] used an ureC gene amplification PCR assay in gastric biopsy specimens and found it more sensitive than histology staining and ^13^C-UBT for diagnosing H. pylori infection. Ramirez-Lazaro et al. [18] showed that real-time PCR was positive for 16 sRNA+urea+23sRNA in 35 (67%) of the 52 histology-negative biopsy specimens, being more sensitive than conventional histology or immunohistochemistry. Most recently, Belda et al. [13] showed that a new real-time PCR system for detecting H. pylori in gastric biopsies was more sensitive than traditional diagnostic methods. In 154 symptomatic patients, the traditional gold standard detected 85 patients (55.19%) with H. pylori infection and the real-time PCR was positive for H. pylori in antral biopsies of 101 patients (65.6%).

Due to significantly high sensitivity, fqPCR detected additional H. pylori infection in 8% of gold standard negative or 10.7% of routine histology-negative gastric mucosa specimens in our study. Zsikla et al. [11] have shown that qualitative nested and quantitative PCR can detect H. pylori in about 20% of histologic-negative gastric biopsies. In patients with upper gastrointestinal bleeding, biopsy-based methods, such as RUT, histology and bacterial culture, have a low sensitivity at 45% to 70% and a high false-negative rate at 30% to 55% [6]. Weiss et al. [19] demonstrated that in gastric biopsy specimens, H. pylori was detected in 29 (52%) of cases by PCR, and only 11 (20%) by CLOtest or immunohistochemical analysis. Ramírez-Lázaro et al. [18] showed that real-time PCR detected H. pylori infection in 67% of histology-negative formalin-fixed paraffin-embedded biopsy samples obtained during peptic ulcer bleeding episodes, while immunohistochemical analysis only detected the infection in only three (6%) of the patients.

Many invasive and non-invasive tests are available to detect H. pylori in children. However, no single test is reliable enough to be the gold standard. Therefore, it is recommended to use concordant results of at least two tests to define the H. pylori infection status [5]. Using this recommended gold standard, the sensitivity and specificity of routine histology, RUT, ^13^C-UBT in our study are excellent and consistent with other reported studies [3,4]. Since our study showed that fqPCR identified significant number of H. pylori infections that would be otherwise missed by histology and conventional assays, gastric mucosa fqPCR is superior to current gold standard for H. pylori detection.

The main limitation of this study is that we did not perform immunohistochemical staining for H. pylori infection in our biopsy samples. Immunohistochemical staining for H. pylori has been shown to be the most sensitive and specific method of staining, and has a low inter-observer variation [20-22]. However, Smith et al. [23] performed a retrospective study to investigate the usefulness of immunohistochemical stains for the diagnosis of H. pylori infection and concluded that the routine use of special stains is not necessary for the identification of H. pylori because the organism is readily identifiable in the majority of cases with H&E staining. It is possible that performing immunohistochemical stains in all our study samples might increase the sensitivity of H. pylori detection, which unlikely would change our conclusion since other studies have shown that PCR-based methods were more sensitive than immunohistochemical analysis [18,19].

Another limitation for gastric mucosa fqPCR is its invasiveness for endoscopic procedure and tissue biopsies. In clinical practice, esophagogastroduodenoscopy examination with tissue biopsy is usually performed to diagnose H. pylori infection in symptomatic children. H&E and special stains are routinely used by pathologist to identify gastritis and H. pylori organisms, in conjunction with RUT. Due to low numbers of H. pylori organisms and minimal gastric inflammation, either histology stain or RUT may not identify H. pylori infection. In this situation, PCR method would accurately diagnose H. pylori infections that would otherwise be missed.

There are reports that analysis of gastric fluids and dental plaques may be used to determine the H. pylori presence [24,25]. Using mucosal fqPCR as a reference, our study showed that gastric fluids and dental plaques fqPCR had sensitivity of 69.7% and 65.2% respectively, much less reliable than gastric mucosa fqPCR assay. However, as non-invasive assays, gastric fluids and dental plaques fqPCR was as sensitive as ^13^C-UBT to detect H. pylori infection. Liu et al. [2] showed that the prevalence of oral H. pylori in dental plaques approximated that of gastric H. pylori in dyspeptic patients. With further studies, fqPCR in dental plaques or gastric fluid obtained from nasogastric tube can be potentially validated as an alternative non-invasive test for H. pylori detection.

Our results also showed that H. pylori infection in children with gastritis varies from 25.9% to 56.5% among different age groups, with 10 to 12 year-old age group at highest risk. However, due to small samples in each age group, the age prevalence of H. pylori infection in children needs to be confirmed by a multi-center study with a larger sample size.

## Conclusion

In conclusion, our study demonstrated that gastric mucosa fqPCR was more sensitive than routine histology, RUT, ^13^C-UBT alone or in combination to detect H. pylori infection in children with chronic gastritis. Either gastric fluid or dental plaque fqPCR is as reliable as ^13^C-UBT for H. pylori detection. Larger prospective and multicenter studies are required to validate our finding that gastric mucosa fqPCR can potentially be established as a new gold standard for H. pylori detection.

## Abbreviations

H. pylori: *Helicobacter pylori*; PCR: Polymer chain reaction; RUT: Rapid urease test; C-UBT: ^13^C-urea breath test; fqPCR: fluorescent quantitative PCR.

## Competing interests

The authors declare no competing interests associated with this manuscript.

## Authors’ contributions

ZO and LX: study concept and design, acquisition of data, analysis and interpretation of data and drafting of the manuscript. LG, LL, PC, MY, YZ, and ZZ: acquisition of data. DL: Critical revision of the manuscript for important intellectual content. HX and SG: study supervision. All authors read and approved the final manuscript.

## Pre-publication history

The pre-publication history for this paper can be accessed here:

http://www.biomedcentral.com/1471-230X/13/7/prepub
